# Cancer Screening Knowledge and Behavior in a Multi-Ethnic Asian Population: The Singapore Community Health Study

**DOI:** 10.3389/fonc.2021.684917

**Published:** 2021-08-12

**Authors:** Tyson Kin-Chung Chan, Linda Wei Lin Tan, Rob M. van Dam, Wei Jie Seow

**Affiliations:** ^1^Preventive Medicine, National University Health System, Singapore, Singapore; ^2^Saw Swee Hock School of Public Health, National University of Singapore and National University Health System, Singapore, Singapore; ^3^Department of Medicine, Yong Loo Lin School of Medicine, National University of Singapore and National University Health System, Singapore, Singapore

**Keywords:** behavior, breast cancer, cancer, cervical cancer, colorectal cancer, disparities, knowledge, screening

## Abstract

**Background:**

Cancer has become the leading cause of mortality in Singapore and among other Asian populations worldwide. Despite the presence of National Cancer Screening programmes in Singapore, less than half of the population has had timely screening according to guidelines. The underlying factors of poor cancer screening rates and health outcomes among Asian ethnic groups remain poorly understood. We therefore examined cancer screening participation rates and screening behavior in a multi-ethnic Singapore population.

**Methods:**

We collected data from 7,125 respondents of the 2015–2016 Singapore Community Health Study. Factors associated with cervical, breast, and colorectal cancer screening were evaluated using modified Poisson regression. Adjusted prevalence ratios were computed with 95% confidence intervals after adjusting for confounders.

**Results:**

The mean age of the respondents was 57.7 ± 10.9 years; 58.9% were female and were predominately Chinese (73.0%), followed by Malay (14.2%), and Indian (10.9%). Less than half of the respondents in the recommended age groups had undergone cancer screening (cervical, 43%; breast, 35.1%; colorectal, 27.3%). Malay respondents were significantly less likely to screen as recommended for cervical (aPR = 0.75, CI = 0.65–0.86, p < 0.001), breast (aPR = 0.83, CI = 0.68–0.99, p = 0.045), and colorectal cancer (aPR = 0.55, CI = 0.44–0.68, p < 0.001), as compared to Chinese respondents. Respondents who had obtained lower secondary level education were 42% more likely to screen for cervical cancer (aPR = 1.42, CI = 1.23–1.64, p < 0.001), and 22% more likely to screen for breast cancer (aPR = 1.22, CI = 1.02–1.46, p = 0.032), compared to those with primary level education and below. Respondents with a household income ≥S$10,000/month were 71% more likely to screen for breast cancer (aPR = 1.71, CI = 1.37–2.13, p < 0.001), as compared with <$2,000/month.

**Conclusions:**

Ethnicity and socio-economic status were significantly associated with lower uptake of cancer screening tests in Singapore. To improve the screening uptake among disadvantaged groups, a multi-faceted approach is needed that addresses the barriers to screening such as the adequacy of subsidy schemes and ethnic differences.

## Introduction

GLOBOCAN estimated 18.1 million new cases and 9.6 million cancer deaths worldwide in 2018 (1). Approximately half of the global burden of cancer was attributed to Asia in part due to 60% of the global population residing there and is projected to continue increasing as life expectancy improves (1). Cancer is the leading cause of mortality among both native and immigrant Asians irrespective of their country of residence (2–6). Those residing in Western countries where they are the ethnic minority are more likely to present with advanced stages of cancer and to have lower cancer screening rates in comparison to non-Hispanic whites (2–6). A study in Canada demonstrated breast cancer screening disparities among immigrant women by world region of origin and found that South Asian women, which included Indians, had the lowest screened as recommended rate at 48.5%. East Asian and Pacific women, which included Chinese, had a screened as recommended rate of 61.1% (7). In another study in the United States, regression models showed that foreign-born women from Southeast Asia, which included Singaporean Chinese, Indian and Malays, were more likely to be unscreened for cervical cancer (13.7%) compared to US-born women (7.6%) (8). Studies conducted in Western countries are often too underpowered to distinguish different Asian ethnic sub-groups (9, 10). Singapore is an opportune country to explore cancer screening behaviors among Asian ethnic sub-groups due to the nation’s large population of East Asians (Chinese), South Asians (Indians), and South East Asians (Malays).

In Singapore, cancer was the leading cause of death with 29.1% of total deaths in 2017 (11, 12). The Singapore Cancer Registry data showed that colorectal cancer (17.2%) had replaced lung cancer (14.8%) to become the most common cancer in men (13). Breast cancer (29.1%) and colorectal cancer (13.4%) remained the most common cancers in women (13). National Cancer Screening programmes have been launched to reduce morbidity and mortality in breast, cervical, and colorectal cancers. Through the Health Promotion Board (HPB), Singapore became the first Asian country to launch a population-wide national breast cancer screening programme in 2002 for females aged 50–69 years (14), which was shortly followed by the launch of a national cervical cancer screening programme in 2004 for females aged 25–69 years (15). From 2003, Singapore Cancer Society has been involved in large-scale opportunistic colorectal cancer screening. In 2011, HPB launched a national screening programme for colorectal cancer for individuals aged 50 and above (16). Although public awareness of screening and accessibility increased, the National Health Survey 2010 data showed that timely screening remained low with less than half of the population having had timely screening according to guidelines (17). Therefore, it is necessary to evaluate the progress of cancer screening.

This study aims to examine cervical, breast, and colorectal cancer screening behaviors in Singapore and identify how socio-demographic factors such as ethnicity and socio-economic status are associated with cancer screening rates. We will also examine the extent of the knowledge–behavior gap in cancer screening behavior. In doing so, we aim to better understand the determinants of cancer screening behaviors in the population of Singapore to improve screening programmes for the under-screened groups.

## Methods

### Study Population and Study Setting

Data used in this cross-sectional study was derived from the Singapore Community Health Study (CHS), a population health survey that was conducted in Queenstown and Bukit Panjang (18, 19) between April 2015 and August 2016. The surveyed districts were catchment areas for the National University Health System and resembled the age, gender, and ethnic distribution of the national population census (20). All Singaporean citizens and permanent residents aged 40 and above were eligible for participation in CHS. A total of 7,125 residents in this age group were interviewed (Bukit Panjang—4,906; Queenstown—2,219).

### Data Collection

Recruitment in CHS occurred through community club events and advertisements (banners/posters) in residential blocks. All household members were eligible to participate in the study, which was voluntary and self-selected. Households also received invitation letters at least two weeks before being visited by a trained interviewer. A group of field work team members were required to pass an assessment after undergoing a minimum of three days of training by qualified staff from the University on consent-taking and administering the questionnaire before they were allowed to interview participants. A response rate could not be ascertained due to the multi-modal recruitment process.

Interviewer-administered standardized questionnaires were conducted in the preferred language and location of the participant (own home or at the nearby Residents’ Committee centre). A translator was arranged if required. Informed consent was taken from all participants.

The questionnaire explored socio-demographics (age, gender, ethnicity), socio-economic indicators (education level, household income, housing type), living arrangement (alone or with others), lifestyle practices including smoking and alcohol consumption, medical history (previous cancer diagnosis or any family history of any cancer) and cancer screening practices. Education level was categorized as primary [passing the Primary School Leaving Examination (PSLE)], lower secondary (years 1–3), secondary (passing the Singapore-Cambridge General Certificate of Education (GCE) Normal or Ordinary Level Examination), junior college (passing the GCE-Advanced Level Examination), polytechnic/arts institution (obtaining a diploma), and university (obtaining a degree, masters or PhD). For cervical cancer screening, the questions were: “Do you know what a Pap smear is?”; “Have you ever had a Pap smear test?”; “How long ago did you have your last smear done?”. For breast cancer screening the questions were: “Do you know what a mammogram is?”; “Have you ever had a mammogram?”, “How long ago did you have your last mammography done?”. Finally, for colorectal cancer screening the questions were: “Have you ever had a blood stool test to determine whether the stool contains blood?”; “How long ago did you have your last blood stool test done?’; “Have you ever had either sigmoidoscopy or colonoscopy, an examination in which a tube is inserted in the rectum to view the colon for signs of cancer or other health problems?”; “How long ago did you have your last sigmoidoscopy or colonoscopy done?”.

According to the screening guidelines of Singapore (21), the frequency of cervical and breast cancer screening was considered done as recommended if women aged 25–69 years reported having a Pap smear every 3 years, and if women aged 50–69 years reported having a mammogram every 2 years, respectively. Colorectal cancer screening was done as recommended if fecal occult blood test (FOBT) was done annually or sigmoidoscopy/colonoscopy was done once every 10 years for individuals aged ≥50 years.

Ethics approval was obtained from the National Health Group Domain Specific Review Board (2015-00095) as well as the National University of Singapore IRB (S-19-340).

### Statistical Analysis

Baseline characteristics were reported as categorical variables and tabulated using proportions for the descriptive analysis. For estimating prevalence ratios in cross-sectional studies, Zou’s method using multivariate modified Poisson regression with robust sandwich variance was chosen as the most viable statistical option as described in Lee’s Practical Guide for Multivariate Analysis of Dichotomous Outcomes (22). This method was utilized to estimate the adjusted prevalence ratios (aPRs) and 95% confidence intervals (CIs) using R packages lmtest v0.9-3.7 and sandwich v2.5-1. Variables identified as determinants of screening behaviors in previous studies (23–28) that proved to be significant in the univariate analysis for the respective cancer groups (*e.g.* age, ethnicity, education, household income, housing type, living arrangement, past history of any cancer, family history of any cancer, and frequent smoking) were used to adjust for potential confounding. The analysis was also stratified by family history. A P-value ≤0.05 was used to determine statistical significance. The knowledge–behavior gap was calculated as the difference in proportions between those that reported having knowledge of the screening test and those that ever did the screening test or screened as recommended. All analysis was performed using R version 3.6.2.

## Results

Respondents of the survey (N = 7,125) were mostly aged 40–69 years (85%) with a mean age of 57.7 ± 10.9 years and ethnically Chinese (73%) with a slight majority of females (58.9%) (**Table 1**). The age, gender, and ethnic distribution of our survey sample resembled the population census during the same time period (**Supplemental Figures 1**–**3**).

**Table 1:** Characteristics of the study population by cancer screening eligibility criteria*.

Characteristic	Total	Cervical Cancer Screening	Breast Cancer Screening	Colorectal Cancer Screening
	N = 7125 n(%)	N = 3584 n(%)	N = 2532 n(%)	N = 5281 n(%)
Age(years)				
40–49	1,842 (25.9)	1058 (29.5)	–	–
50–59	2,386 (33.5)	1447 (40.4)	1449 (57.2)	2384 (45.1)
60–69	1,830 (25.7)	1079 (30.1)	1083 (42.8)	1830 (34.7)
70–79	827 (11.6)	–	–	827 (15.7)
80 and above	240 (3.4)	–	–	240 (4.5)
Gender				
Female	4,197 (58.9)	–	–	3,135 (59.4)
Male	2,928 (41.1)	–	–	2,146 (40.6)
Ethnicity				
Chinese	5,203 (73.0)	2,584 (72.1)	1,893 (74.8)	4,029 (76.3)
Malay	1,014 (14.2)	563 (15.7)	381 (15.0)	720 (13.6)
Indian	777 (10.9)	371 (10.4)	231 (9.1)	473 (9.0)
Others	131 (1.8)	66 (1.8)	27 (1.1)	59 (1.1)
Education				
Primary and below	2,415 (33.9)	1,149 (32.1)	993 (39.2)	2,163 (41.0)
Lower secondary	1,414 (19.8)	705 (19.7)	552 (21.8)	1,176 (22.3)
Secondary	1,546 (21.7)	900 (25.1)	615 (24.3)	1,092 (20.7)
Junior College	391 (5.5)	182 (5.1)	102 (4.0)	247 (4.7)
Polytechnic/Arts Institution	637 (8.9)	309 (8.6)	143 (5.6)	320 (6.1)
University & above	719 (10.1)	338 (9.4)	126 (5.0)	280 (5.3)
Monthly household income ($S)				
<$2,000	2,185 (30.7)	937 (26.1)	754 (29.8)	1,882 (35.6)
$2,000–$3,999	1,586 (22.3)	845 (23.6)	534 (21.1)	1,069 (20.2)
$4,000–$5,999	953 (13.4)	511 (14.3)	316 (12.5)	590 (11.2)
$6,000–$9,999	734 (10.3)	380 (10.6)	205 (8.1)	400 (7.6)
≥$10,000	343 (4.8)	173 (4.8)	114 (4.5)	206 (3.9)
Housing type				
≤2-room public flat	384 (5.4)	156 (4.4)	113 (4.5)	308 (5.8)
3-room public flat	1,795 (25.2)	812 (22.7)	545 (21.5)	1,297 (24.6)
≥4-room public flat/private	4,945 (69.4)	2,615 (73.0)	1,873 (74.0)	3,675 (69.6)
Living arrangement				
Alone	399 (5.6)	162 (4.5)	138 (5.5)	352 (6.7)
With others	6,722 (94.3)	3,420 (95.4)	2,394 (94.5)	4,927 (93.3)
Past history of any cancer				
No	6,867 (96.4)	3,441 (96.0)	2,405 (95.0)	5,044 (95.5)
Yes	258 (3.6)	143 (4.0)	127 (5.0)	237 (4.5)
Family history of any cancer				
No	4,867 (68.3)	2,344 (65.4)	1,602 (63.3)	3,551 (67.2)
Yes	2,258 (31.7)	1,240 (34.6)	930 (36.7)	1,730 (32.8)
Frequent smoking^a^				
No	5,834 (81.9)	3,333 (93.0)	2,401 (94.8)	4,401 (83.3)
Yes	805 (11.3)	102 (2.8)	55 (2.2)	546 (10.3)
Frequent alcohol intake^b^				
No	4,931 (69.2)	2,762 (77.1)	1,961 (77.4)	3,605 (68.3)
Yes	559 (7.8)	134 (3.7)	86 (3.4)	403 (7.6)

^*^Based on recommended screening guidelines for selected cancers as defined by MOH guidelines: cervical cancer—Pap smear for sexually active females aged 25 to 69 years at least once every 3 years; breast cancer—mammography for females aged 50 to 69 years every 2 years; colorectal cancer—fecal occult blood test (FOBT) done annually or sigmoidoscopy/colonoscopy once every 10 years for individuals aged ≥50 years.

a Frequent smoking is defined as smoking cigarettes daily.

b Frequent alcohol intake is defined as having at least 1–4 servings per week.

A majority of the screening-eligible female respondents reported having knowledge of Pap smear (80.0%) and mammography (93.6%). At least three quarters had ever been screened (cervical, 77.2%; breast, 75.2%); whereas, less than half had undergone screening as recommended (cervical, 43.0%; breast, 35.1%) (**Table 2**).

**Table 2:** Cancer screening test knowledge and participation rates.

	Number of respondents eligible for screening as recommended	Reported having knowledge of screening test^╤^	Those who had ever been screened	Those who had screened as recommended*
	Total (N)	n(%)	n(%)	n(%)
Pap Smear	3,584	2,872 (80.0)	2,763 (77.2)	1,539 (43.0)
Mammography	2,532	2,370 (93.6)	1,903 (75.2)	889 (35.1)
FOBT only	5,281	–	2,267 (42.9)	–
Colonoscopy/Sigmoidoscopy only		–	1,167 (22.1)	–
FOBT/Colonoscopy/Sigmoidoscopy		–	2,589 (49.0)	1,440 (27.3)
All of the above°	2,536	–	–	272 (10.7)

^*^Based on recommended screening guidelines for selected cancers as defined by MOH guidelines:

cervical cancer—Pap smear for sexually active females aged 25 to 69 years at least once every 3 years; breast cancer—mammography for females aged 50 to 69 years every 2 years; colorectal cancer—faecal occult blood test (FOBT) done annually or sigmoidoscopy/colonoscopy once every 10 years for individuals aged ≥50 years.

^╤^Due to limitations of the collected data, knowledge for colorectal cancer screening was not reported.

°Pap smear, mammography, and either FOBT or colonoscopy/sigmoidoscopy.

Nearly half of the eligible respondents (49.0%) had ever been screened for colorectal cancer, but only 27.3% had screened within the recommended time period. More respondents had ever had FOBT (42.9%) compared to colonoscopy or sigmoidoscopy (22.1%). Among female respondents aged 50–69 years, only 10.7% had screened for all three cancers (cervical, breast, colorectal) within the recommended time period.

### Characteristics Associated With Female Cancer Screening (Cervical and Breast) Knowledge of Screening Test

In the multivariate analysis, Malay and Indian ethnicity and higher level of education were significantly associated with reporting having knowledge of the Pap smear test (**Table 3**).

**Table 3:** Adjusted prevalence ratio (aPR) estimates for characteristics associated with knowledge of and participation in cervical and breast cancer screening.

Characteristic	Cervical cancer^a^						Breast cancer^b^					
	Reported having knowledge of the screening test		Those who had ever been screened		Those who had screened as recommended*		Reported having knowledge of the screening test		Those who had ever been screened		Those who had screened as recommended**	
	aPR (95% CI)	p-value	aPR (95% CI)	p-value	aPR (95% CI)	p-value	aPR (95% CI)	p-value	aPR (95% CI)	p-value	aPR (95% CI)	p-value
Age (years)
40–49	Ref		Ref		Ref	
50–59	1.01 (0.98–1.05)	0.50	1.00 (0.95–1.04)	0.92	0.91 (0.83–0.99)	0.037	Ref		Ref		Ref	
60–69	0.96 (0.91–1.01)	0.086	0.98 (0.93–1.04)	0.53	0.73 (0.64–0.83)	<0.001	1.00 (0.98–1.03)	0.73	1.13 (1.07–1.19)	<0.001	0.99 (0.87–1.13)	0.86
Ethnicity
Chinese	Ref		Ref		Ref		Ref		Ref		Ref
Malay	1.17 (1.12–1.22)	<0.001	0.97 (0.91–1.02)	0.26	0.75 (0.65–0.86)	<0.001	0.92 (0.88–0.96)	<0.001	0.79 (0.72–0.87)	<0.001	0.83 (0.68–0.99)	0.045
Indian	1.18 (1.13–1.23)	<0.001	1.03 (0.97–1.09)	0.40	1.03 (0.92–1.17)	0.59	0.99 (0.95–1.03)	0.61	0.94 (0.86–1.03)	0.20	1.03 (0.85–1.25)	0.74
Others	1.02 (0.93–1.12)	0.68	1.02 (0.92–1.15)	0.67	0.89 (0.69–1.16)	0.40	0.86 (0.73–1.02)	0.075	1.01 (0.83–1.22)	0.96	1.12 (0.71–1.75)	0.63
Education						
Primary and below	Ref		Ref		Ref		Ref		Ref		Ref
Lower secondary	1.27 (1.18–1.36)	<0.001	1.22 (1.15–1.31)	<0.001	1.42 (1.23–1.64)	<0.001	1.07 (1.03–1.11)	<0.001	1.10 (1.02–1.19)	0.009	1.22 (1.02–1.46)	0.032
Secondary	1.47 (1.38–1.56)	<0.001	1.21 (1.14–1.29)	<0.001	1.40 (1.22–1.60)	<0.001	1.10 (1.07–1.14)	<0.001	1.13 (1.06–1.21)	<0.001	1.33 (1.12–1.57)	0.001
Junior College	1.43 (1.33–1.55)	<0.001	1.19 (1.09–1.30)	<0.001	1.30 (1.07–1.59)	0.009	1.11 (1.07–1.15)	<0.001	1.09 (0.97–1.22)	0.14	1.10 (0.82–1.48)	0.51
Polytechnic	1.48 (1.38–1.59)	<0.001	1.15 (1.06–1.25)	0.001	1.34 (1.14–1.58)	0.001	1.11 (1.07–1.15)	<0.001	1.15 (1.05–1.26)	0.004	1.19 (0.92–1.54)	0.19
University	1.48 (1.38–1.59)	<0.001	1.16 (1.07–1.26)	<0.001	1.44 (1.22–1.70)	<0.001	1.11 (1.07–1.16)	<0.001	1.15 (1.05–1.28)	0.005	1.31 (1.01–1.68)	0.039
Monthly household income ($S)						
<$2,000	Ref		Ref		Ref		Ref		Ref		Ref
$2,000–$3,999	1.05 (1.00–1.11)	0.067	1.10 (1.04–1.17)	0.002	1.21 (1.06–1.37)	0.004	1.00 (0.97–1.04)	0.81	1.04 (0.98–1.12)	0.22	1.12 (0.95–1.32)	0.18
$4,000–$5,999	1.10 (1.04–1.16)	0.001	1.16 (1.09–1.24)	<0.001	1.28 (1.12–1.47)	<0.001	1.01 (0.98–1.05)	0.4	1.07 (0.99–1.16)	0.067	1.25 (1.03–1.50)	0.02
$6,000–$9,999	1.10 (1.05–1.17)	<0.001	1.20 (1.12–1.28)	<0.001	1.48 (1.29–1.71)	<0.001	1.00 (0.96–1.04)	0.98	1.02 (0.93–1.12)	0.63	1.18 (0.95–1.47)	0.14
≥$10,000	1.12 (1.06–1.19)	<0.001	1.25 (1.16–1.34)	<0.001	1.51 (1.28–1.79)	<0.001	1.01 (0.98–1.05)	0.41	1.20 (1.11–1.30)	<0.001	1.71 (1.37–2.13)	<0.001
Housing type						
≤2-room public flat	Ref		Ref		Ref		Ref		Ref		Ref
3-room public flat	1.06 (0.93–1.20)	0.41	1.03 (0.88–1.20)	0.74	1.05 (0.79–1.40)	0.78	1.05 (0.97–1.14)	0.26	1.27 (1.05–1.53)	0.013	1.22 (0.84–1.77)	0.30
≥4-room public flat/private	1.22 (1.07–1.38)	0.003	1.20 (1.04–1.39)	0.013	1.19 (0.90–1.57)	0.31	1.06 (0.98–1.15)	0.14	1.36 (1.13–1.63)	0.001	1.30 (0.91–1.87)	0.15
Living arrangement						
Alone	Ref		Ref		Ref		–		–	–	–	–
With others	0.99 (0.90–1.09)	0.86	1.30 (1.11–1.53)	0.001	1.81 (1.31–2.52)	0.002	–		–	–	–	–
Past history of any cancer
No	–		–	–	–	–	Ref		Ref		Ref	
Yes	–		–	–	–	–	1.02 (0.98–1.05)	0.44	1.16 (1.08–1.24)	<0.001	1.69 (1.41–2.02)	<0.001

a Multivariate modified Poisson regression model analyses were adjusted for age, ethnicity, education, monthly household income, housing type, and living arrangement.

b Multivariate modified Poisson regression model analyses were adjusted for age, ethnicity, education, monthly household income, housing type, and past history of any cancer. No significant characteristics were found to be associated with knowledge of mammography on univariate analysis.

*Based on recommended screening guideline for cervical cancers as defined by MOH guidelines: Pap smear for sexually active females aged 25 to 69 years at least once every 3 years.

**Based on recommended screening guideline for breast cancer as defined by MOH guidelines: mammography for females aged 50 to 69 years every 2 years.

Individuals of Malay (aPR = 1.17, CI = 1.12–1.22, p < 0.001) and Indian (aPR = 1.18, CI = 1.13–1.23, p < 0.001) ethnicity were more likely to report knowledge of Pap smear testing as compared with ethnic Chinese. In contrast, Malay women were less likely than Chinese women to report having knowledge of mammography (aPR = 0.92, CI = 0.88–0.96, p < 0.001) (**Table 3**).

All levels of education higher than primary school and below were significantly associated with self-reported knowledge of the screening tests even for those with only lower secondary school education. Compared with having attained at most primary school education, the prevalence of self-reported knowledge regarding Pap smear was already 47% higher at secondary school level education (aPR = 1.47, CI = 1.38–1.56, p < 0.001). Household income and housing type showed weaker associations with self-reported Pap smear knowledge.

### Ever Screened

Education level and household income were significantly associated with ever having a Pap smear test (**Table 3**). In addition, women living with others (aPR = 1.30, CI = 1.11–1.53, p = 0.001) were 30% more likely to ever have a Pap smear compared with those living alone. Older age, higher education level, high household income, and having a more expensive housing type were significantly associated with ever having a mammogram, whereas Malay ethnicity was associated with a lower likelihood of ever having a mammogram (**Table 3**).

Among those who reported no knowledge of the screening tests (N = 711 for Pap smear; N = 161 for mammogram), 44.7% underwent screening with Pap smear (n = 318) and 26.1% with mammogram (n = 42). For Pap smear, respondents of Malay (aPR = 0.45, CI = 0.27–0.75, p = 0.002) and Indian (aPR = 0.36, CI = 0.16–0.82, p = 0.015) ethnicity were less likely to report this behavior compared to Chinese (**Supplemental Table 1**). The sub-group analysis was not reported for mammogram due to the small sample size.

### Screened as Recommended

Participants of Malay ethnicity (aPR = 0.75, CI = 0.65–0.86, p < 0.001) and those aged 60–69 years (aPR = 0.73, CI = 0.64–0.83, p < 0.001) were significantly less likely to undergo Pap smear screening as recommended at least once every three years (**Table 3**). Socio-economic factors directly associated with screening as recommended were higher education level and higher household income. Respondents living with others (aPR = 1.81, CI = 1.31–2.52, p = 0.002) were 81% more likely to screen as recommended compared to those living alone. Similar to cervical cancer screening, Malay ethnicity (aPR = 0.83, CI = 0.68–0.99, p = 0.045) was observed to be less likely to screen for breast cancer as recommended compared to Chinese. Higher education and higher household income were also significantly associated with mammogram screening as recommended at least once every two years (**Table 3**). A higher proportion of respondents reported desirable cancer screening behavior among those who had any family history of any cancer in comparison with those without any family history (**Supplementary Table 4**).

### Characteristics Associated With Colorectal Cancer Screening

Older age (60–79 years), higher education level, higher household income, past history of any cancer, and family history of any cancer were significantly associated with having ever screened for colorectal cancer by FOBT and/or scope (colonoscopy/sigmoidoscopy) (**Table 4**). Malay and Indian respondents as well as those who smoked daily were significantly less likely to be ever screened. The same variables that were significantly associated with having ever been screened by FOBT, colonoscopy, or sigmoidoscopy were also significantly associated with screening as recommended (**Table 4**).

**Table 4:** Adjusted prevalence ratio (aPR) estimates for characteristics associated with participation in colorectal cancer screening.

Characteristic	Those who had ever been screened by scope		Those who had ever been screened by FOBT		Those who had ever been screened by any three colorectal cancer tests		Those who had screened as recommended*	
	aPR (95% CI)	p-value	aPR (95% CI)	p-value	aPR (95% CI)	p-value	aPR (95% CI)	p-value
Age (years)								
50–59	Ref		Ref		Ref		Ref	
60–69	1.31 (1.14–1.50)	<0.001	1.16 (1.07–1.26)	<0.001	1.13 (1.06–1.22)	0.001	1.25 (1.12–1.40)	<0.001
70–79	1.55 (1.27–1.88)	<0.001	1.19 (1.05–1.34)	0.005	1.17 (1.05–1.30)	0.004	1.34 (1.14–1.59)	0.001
80 and above	1.60 (1.19–2.14)	0.002	1.17 (0.96–1.42)	0.12	1.20 (1.02–1.42)	0.032	1.14 (0.85–1.53)	0.38
Gender								
Female	Ref		Ref		Ref		Ref	
Male	1.11 (0.98–1.25)	0.10	0.98 (0.91–1.06)	0.70	1.02 (0.95–1.09)	0.54	1.08 (0.97–1.21)	0.14
Ethnicity								
Chinese	Ref		Ref		Ref		Ref	
Malay	0.51 (0.39–0.66)	<0.001	0.50 (0.42–0.58)	<0.001	0.50 (0.43–0.58)	<0.001	0.55 (0.44–0.68)	<0.001
Indian	0.78 (0.63–0.98)	0.034	0.73 (0.63–0.84)	<0.001	0.78 (0.68–0.88)	<0.001	0.92 (0.77–1.10)	0.36
Others	0.72 (0.42–1.23)	0.23	0.67 (0.47–0.97)	0.032	0.67 (0.48–0.92)	0.015	0.75 (0.48–1.19)	0.22
Education								
Primary and below	Ref		Ref		Ref		Ref	
Lower secondary	1.00 (0.84–1.19)	0.98	0.99 (0.89–1.11)	0.89	0.99 (0.90–1.09)	0.78	1.07 (0.92–1.24)	0.38
Secondary	1.32 (1.11–1.57)	0.002	1.20 (1.08–1.33)	<0.001	1.22 (1.12–1.33)	<0.001	1.25 (1.07–1.44)	0.004
Junior College	1.27 (0.99–1.63)	0.062	1.12 (0.95–1.31)	0.17	1.11 (0.96–1.28)	0.16	1.15 (0.92–1.44)	0.23
Polytechnic/Arts Institution	1.45 (1.16–1.81)	0.001	1.36 (1.19–1.55)	<0.001	1.33 (1.19–1.49)	<0.001	1.46 (1.21–1.76)	<0.001
University & above	1.41 (1.09–1.82)	0.008	1.37 (1.19–1.58)	<0.001	1.30 (1.14–1.48)	<0.001	1.41 (1.14–1.74)	0.002
Monthly household income ($S)								
<$2,000	Ref		Ref		Ref		Ref	
$2,000–$3,999	1.00 (0.84–1.18)	0.98	1.06 (0.96–1.17)	0.22	1.06 (0.97–1.15)	0.19	1.08 (0.94–1.25)	0.26
$4,000–$5,999	1.16 (0.96–1.40)	0.14	0.98 (0.87–1.11)	0.77	1.01 (0.91–1.13)	0.78	1.10 (0.94–1.30)	0.25
$6,000–$9,999	1.22 (0.98–1.51)	0.069	1.04 (0.91–1.18)	0.59	1.07 (0.96–1.20)	0.23	1.18 (0.98–1.41)	0.074
≥$10,000	1.47 (1.14–1.90)	0.003	1.20 (1.04–1.39)	0.013	1.18 (1.03–1.35)	0.014	1.30 (1.04–1.62)	0.021
Housing type								
≤2-room public flat	Ref		Ref		Ref		Ref	
3-room public flat	1.10 (0.82–1.46)	0.53	1.04 (0.86–1.26)	0.69	1.06 (0.89–1.25)	0.52	1.09 (0.84–1.42)	0.50
≥4-room public flat/private	1.10 (0.83–1.45)	0.50	1.21 (1.01–1.45)	0.038	1.18 (1.00–1.38)	0.049	1.24 (0.97–1.59)	0.093
Past history of any cancer								
No	Ref		Ref		Ref		Ref	
Yes	2.06 (1.74–2.45)	<0.001	1.20 (1.04–1.39)	0.013	1.36 (1.23–1.52)	<0.001	1.53 (1.28–1.84)	<0.001
Family history of any cancer								
No	Ref		Ref		Ref		Ref	
Yes	1.25 (1.11–1.42)	<0.001	1.08 (1.00–1.16)	0.048	1.10 (1.03–1.18)	0.004	1.20 (1.08–1.33)	0.001
Frequent smoking^a^								
No	Ref		Ref		Ref		Ref	
Yes	0.67 (0.52–0.87)	0.002	0.71 (0.60–0.83)	<0.001	0.73 (0.63–0.84)	<0.001	0.73 (0.59–0.90)	0.003

Multivariate modified Poisson regression model analyses were adjusted for age, gender, ethnicity, education, monthly household income, housing type, past history of any cancer, family history of any cancer, and frequent smoking.

*Based on recommended screening guidelines for colorectal cancer as defined by MOH guidelines:

colorectal cancer—faecal occult blood test (FOBT) done annually or sigmoidoscopy/colonoscopy once every 10 years for individuals aged ≥50 years.

a Frequent smoking is defined as smoking cigarettes daily.

A key difference was that among the ethnic groups, only Malay ethnicity (aPR = 0.55, CI = 0.44–0.68, p < 0.001), and not Indian ethnicity, remained significantly associated with a lower likelihood of screening as recommended.

We examined determinants of screening as recommended for all three cancers among eligible women aged 50–69. Higher level of education and higher household income were significantly associated with having screened as recommended for all three cancers, whereas Malay ethnicity (aPR = 0.53, CI = 0.33–0.84, p = 0.008) was significantly associated with a lower likelihood as compared with Chinese ethnicity (**Supplemental Table 2**).

### Knowledge–Behaviour Gap

**Figure 1:**
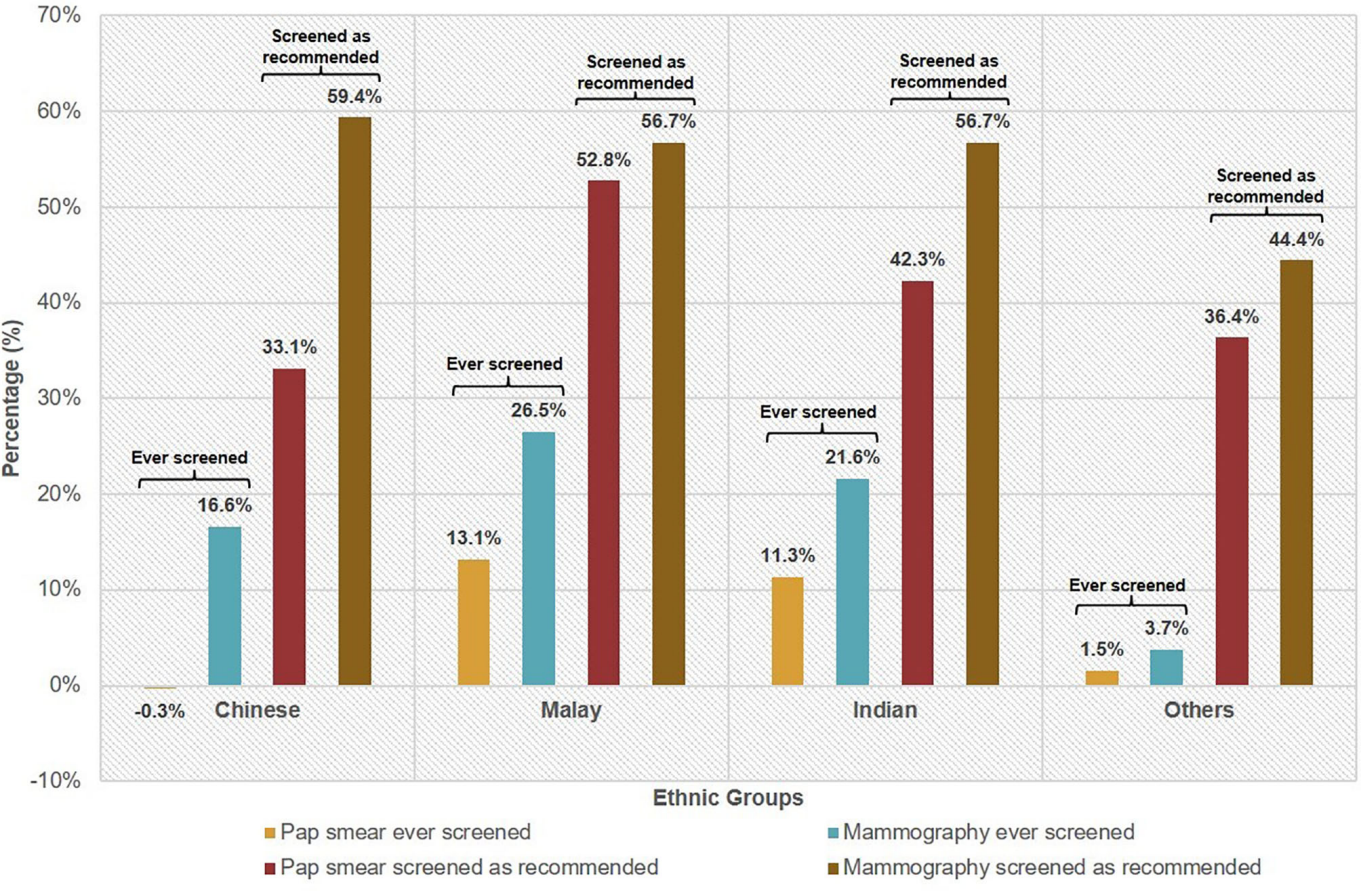
Knowledge–behaviour gap^╤^ of female cancer screening* by ethnicity. ^╤^Knowledge–behaviour gap is defined as the difference in proportions between those that reported having knowledge of the screening test and those that ever did the screening test or screened as recommended. *Based on recommended screening guidelines for selected cancers as defined by MOH guidelines: cervical cancer—Pap smear for sexually active females aged 25 to 69 years at least once every 3 years; breast cancer—mammography for females aged 50 to 69 years every 2 years.

The gap between the percentage that reported knowledge of Pap smear and were ever screened with Pap smear was 2.8% (**Table 2**). For mammography, the gap was higher at 18.4%. Our multivariate analysis indicated the Malay ethnicity was in general less likely to exhibit cancer screening behavior compared with ethnic Chinese. The knowledge–behavior gap among the ethnicities was calculated using the difference in proportions between those that reported having knowledge of the screening test and those that ever did the screening test or screened as recommended. For ever having done the screening test, Malays had the largest knowledge–behavior gap with 13.1% for Pap smear and 26.5% for mammography (**Figure 1**).

Likewise, Malays exhibited the largest knowledge–behavior gap at 52.8% for having screened with Pap smear as recommended. For having screened with mammography as recommended, the gaps were similarly high across the three ethnicities—Chinese (59.4%), Malay (56.7%), Indian (56.7%).

## Discussion

Although screening recommendation guidelines vary slightly between countries, our screened as recommended participation rates fell behind other high-income East Asian countries such as Taiwan in 2016 (cervical, 72.1%; breast, 39.3%; colorectal, 40.7%) (29), and South Korea in 2014 (cervical, 66.1%; breast, 66.0%; colorectal, 29.1%) (30). We also performed poorer compared to Western countries such as the United States in 2015 (cervical, 81%; breast, 71.6%; colorectal, 62.9%) (31) and the United Kingdom in 2017/18 (cervical, 71.4%; breast, 71.1%; colorectal, 57.7%) (32).

Compared to the cancer screening participation rates measured in the 2004 and 2010 national health surveys (17, 33), our screened as recommended participation rates did not indicate significant improvements (**Supplemental Table 3**). For example, the proportion of women who had gone for mammogram as recommended was 35.1% in our study, down from 39.6% in 2010. The proportion of Singapore residents who underwent colorectal screening as recommended was 27.3% in our study, up from 20.2% in 2010. Although the health promotion efforts over the years may have resulted in only modest changes in the screened as recommended participation rates, it is reassuring to observe that between 2004 and 2016, the ever screened rates have seen an upward trend (cervical, 70.1 *vs* 77.1%; breast, 54.2 *vs* 75.2%) in tandem with a downward trend in the size of the knowledge–behavior gap (cervical, 10.7 *vs* 2.8%; breast, 25.7 *vs* 18.4%). Improvements were also seen in colorectal cancer screening participation rates between 2004 and 2016 in ever screened with FOBT (17.3 *vs* 42.9%) and ever screened with sigmoidoscopy/colonoscopy (11.2 *vs* 22.1%).

Our results demonstrate that screening knowledge and behaviors differ substantially by socio-economic status and ethnicity in Singapore. Higher educational level and household income were found to be significantly associated with screening as recommended for cervical, breast, and colorectal cancers. Malay ethnicity was associated with a lower likelihood of screening as recommended as compared with Chinese ethnicity. Cancer screening disparities associated with socio-economic status and ethnicity were reported in previous studies in Singapore (25–27, 34–38), as well as internationally (39, 40). However, limitations to the existing local literature include small sample sizes of the Malay and Indian ethnic minorities with oversampling of the Chinese ethnic majority, assessment of a single cancer screening modality, and age of the data. These limit the ability to generalize findings to the population and develop targeted population health interventions. Our study attempts to better estimate the true population effect sizes through our large representative sample size of 5,203 Chinese, 1,014 Malay, and 777 Indian respondents in the community setting.

Over the years, the Singapore Ministry of Health has endeavored to address the need to improve cancer screening participation rates, which culminated in the 2017 launch of the Enhanced Screen for Life Programme by the Health Promotion Board. This enabled eligible Singaporeans to screen for cervical, breast, and colorectal cancer from as low as $0–$5 per screening visit (41). Although affordability is an important consideration to address the socio-economic disparities, the continued low participation rates suggest there are additional barriers to address. A survey conducted at four polyclinics in Singapore reported that the most commonly cited reasons for not attending breast cancer screening programmes were lack of any breast problems, lack of time, and fear of pain (37). Another local mixed-methods study on barriers to breast and cervical cancer screening reported that fear of unnecessary treatments, ineffective treatments for early stage cancer, and low test sensitivity for early stage cancer were barriers to screening (28).

The proportion of those reporting having a family history of cancer was similar across cervical, breast, and colorectal cancer screening respondents; however, the association between a positive family history of cancer and cancer screening was only found to be significant among colorectal cancer screening respondents. While other studies have also reported this association among Asian women (26, 42), local screening rates particularly among the higher risk first degree relatives of colorectal cancer patients continue to be low (43, 44). Barriers include poor understanding of the screening guidelines, lack of health promotion messaging by healthcare professionals, fear of the test and the diagnosis, scheduling difficulties, feeling invulnerable since young and asymptomatic, unawareness of genetic risk, and the high cost of colonoscopy (43–45). Risk perception should be emphasized in health promotion messaging among Asian ethnicities as perceived susceptibility to breast, cervical, and colorectal cancers was found to be the lowest among Asian women as compared with White, African American, and Latino women (42).

Observing past studies in tandem with our current study, there is a repetitive trend of Malay ethnicity being less likely to participate in cancer screening when compared to the Chinese ethnic majority and their Indian counterparts (17, 26, 27, 33, 46, 47). For female cancer screening, this may be partly explained by the knowledge–behavior gap demonstrated in our study. This gap may be linked to cultural beliefs among Asian women, which should be appropriately understood in order to craft effective policies and health promotion messages. Previous studies have reported cancer screening barriers related to social stigma, personal modesty, fatalistic attitudes, beliefs that breast cancer is a Western women-affliction, beliefs that mammograms cause cancer, and a preference to be unaware of a fatal disease diagnosis to postpone accompanying fears (28, 34, 37, 48–52). However, these findings are limited to predominantly Chinese respondents. In the neighboring country Malaysia with a high proportion of ethnic Malays, their National Health & Morbidity Survey in 2006 showed that only 7.9% of eligible women had underwent mammography as recommended, and only 12.8% had underwent Pap smear as recommended in 2011 (53). Malaysian studies have reported that Malay women are apprehensive about doing Pap smears especially if they are single or unmarried as it indicates sexual activity. A woman’s partner or family members also hold great influence over decisions to screen due to strong family ties. Lack of knowledge among partners and male family members as well as perceived inaccessibility to a female health-care provider are commonly reported barriers (54–56). Similarly, the presence of male technicians/radiographers was found to be a barrier to mammogram screening (57).

The difference in the knowledge–behavior gap between ethnicities alludes to potential health literacy issues related to language barriers in Pap smear testing. Limited English proficiency and low health literacy among Asian women have been identified as barriers to cancer screening in several international studies where English is the predominant language (58–63). We also observed the phenomenon of Chinese women proceeding with Pap smear testing, despite not being fully aware of the purpose of the test. This may be linked to high trust among Chinese women towards their primary physician, which was reported by a study among Redhill residents in Singapore who were predominantly Chinese. Over half of the respondents rated trust towards primary care doctors and the medical profession as high or very high (64), which has been supported by other studies that reported high regard towards general practitioners in the Asian context (65, 66).

In our study, the knowledge–behavior gap was higher for mammography (18.4%) than for Pap smear (2.8%). Previous studies have suggested logistical and operational issues as reasons for the difference between uptake of Pap smear *versus* mammograms (34, 51, 52). The widespread availability of Pap smear tests as a bedside procedure in general practice clinics has made it readily accessible in contrast to the limited availability of mammography. In addition, most patients are able to state preferences or choose female doctors to perform the Pap smears; however, there is no freedom of choice for radiographer doing the mammograms. Having a male radiographer has been shown to be a barrier to screening in both Western and Asian cultures (67–70).

Strategies to further narrow the knowledge–behavior gap should include developing tailored cancer screening promotion campaigns for the Malay ethnic group, which can be done in close consultation with employers, religious, and community authorities to ensure the messages stay culturally relevant (71–77). To further incentivize cancer screening behavior, we must inculcate a culture of cancer screening through community screening initiatives so that they are seen as a form of social event (71, 78, 79). Targeted and frequent mass media campaigns have been shown to be effective in increasing awareness and compliance for cancer screening (71, 80, 81) as well as being frequently exposed to reminders with cues to action (23, 24, 71, 82, 83). Addressing polyclinic proximity and screening appointment logistics may contribute to improving mammography uptake (51). Further studies will need to be done on Malay-specific barriers and facilitators for screening across the three screening modalities as our analysis showed that only 10.7% screened as recommended for all three, and Malays had a higher propensity to not be screened. Existing studies in Singapore had predominantly Chinese respondents and focused on specific screening modalities (23–28). Further studies comparing cancer screening knowledge and behavior between Malays residing in Singapore *versus* Malays residing in Malaysia would help to elicit environmental and cultural influences.

### Strengths and Limitations of the Study

Strengths of this study include a large sample population that resembles the overall age, ethnic, and gender distribution of the Singapore population (**Supplemental Figures S1**–**S3**) (84). Self-selection bias was minimized through the use of a door-to-door recruitment strategy. Misclassification due to interviewer bias, social desirability bias, or recall bias was reduced through the use of a standardized questionnaire consisting of closed-ended, easy to understand questions, simple response options, and trained interviewers that followed the designed question and answer format strictly. However, there are a few limitations to our study. As our survey questions were modelled after the National Health Survey to allow for comparisons, the questions did not differentiate whether the tests were done for screening or diagnostic purposes. In addition, the questions did not differentiate if the participant was screening regularly as recommended or had coincidentally last screened in the recommended time period. As a result, the reported screened as recommended participation rates may be an overestimation of the true value. We were unable to corroborate the self-reported cancer screening data with objective data from medical databases. Another limitation was the inclusion criteria due to the interest of regional health system in targeting interventions on those aged 40 and above in their catchment area, which meant the cervical cancer screening age group from 25 to 39 years was unrepresented. Due to this targeted population, all household members who met the inclusion criteria were included in the Community Health Study; however, data on the proportion of households with more than one member who participated in the study were not available, and statistical analysis adjusting for such potential clustering effects was not performed.

## Conclusions

Cancer screening knowledge and behaviors differ substantially between Asian ethnic sub-groups even within the confines of the island state of Singapore. Asian ethnicity represents a heterogeneous group with different religious and cultural traditions, and our results suggest that it is important to distinguish different ethnic sub-groups in future studies of screening behavior. Ethnic Malays are therefore, a key target population for further research and interventions to narrow the knowledge–behavior gap. Design of targeted cancer screening programmes and health promotion messaging by healthcare providers should include sensitivity to ethnic differences as well as female-specific cancer screening facilitators and barriers, which will help to further increase the uptake of cancer screening. The population-based cancer screening programmes are essential to Singapore’s preventive health strategy. The availability of subsidized rates has allowed more members of the population to access cancer screening, but the overall cancer screening rates still remain low. Socio-economic factors such as educational and income level remain important aspects that policy makers and healthcare organizations should address to improve cancer screening.

## Data Availability Statement

The datasets used in this article are available from the corresponding authors on reasonable request.

## Ethics Statement

The studies involving human participants were reviewed and approved by National Health Group Domain Specific Review Board (2015-00095) and the National University of Singapore IRB (S-19-340). The patients/participants provided their written informed consent to participate in this study.

## Author Contributions

TC participated in the design of this study, performed the statistical analysis, interpreted the data, and drafted the manuscript. LT participated in the design of the Community Health Study, coordination, and data collection. RD is the principal investigator of the Community Health Study and participated in the manuscript revision of this study. WJS participated in the design of this study, the statistical analysis, interpretation of the data, and the manuscript revision. All authors contributed to the article and approved the submitted version.

## Funding

The publication of this report was funded by the National University of Singapore Start-Up and the Ministry of Education Tier 1 grants. The Community Health Study was supported by grants from the Ministry of Health, Singapore, National University of Singapore and National University Health System, Singapore. The funding bodies had no role in the interpretation of the data and the formulation of this report.

## Conflict of Interest

The authors declare that the research was conducted in the absence of any commercial or financial relationships that could be construed as a potential conflict of interest.

## Publisher’s Note

All claims expressed in this article are solely those of the authors and do not necessarily represent those of their affiliated organizations, or those of the publisher, the editors and the reviewers. Any product that may be evaluated in this article, or claim that may be made by its manufacturer, is not guaranteed or endorsed by the publisher.

## Supplementary Material

Supplementary Table 1 Adjusted prevalence ratio (aPR) estimates for characteristics associated with those tested without knowledge of Pap smear. Multivariate modified Poisson regression model analyses were adjusted for age, ethnicity, education, monthly household income, housing type, and living arrangement. *Based on recommended screening guideline for cervical cancers as defined by MOH guidelines: Pap smear for sexually active females aged 25 to 69 years at least once every 3 years.

Supplementary Table 2 Adjusted prevalence ratio (aPR) estimates for characteristics associated with screening as recommended for all three cancers. Multivariate modified Poisson regression model analyses were adjusted for age, ethnicity, education, monthly household income, housing type, living arrangement, past history of any cancer, family history of any cancer, and frequent smoking. *Based on recommended screening guidelines for selected cancers as defined by MOH guidelines: cervical cancer—Pap smear for sexually active females aged 25 to 69 years at least once every 3 years; breast cancer—mammography for females aged 50 to 69 years every 2 years; colorectal cancer–faecal occult blood test (FOBT) done annually or sigmoidoscopy/colonoscopy once every 10 years for individuals aged ≥50 years. ^a^Frequent smoking is defined as smoking cigarettes daily.

Supplementary Table 3 Cancer screening participation rates in Singapore. NHS National Health Survey; CHS, Community Health Survey. Unless otherwise stated, the screening questions involved age groups 25–69 for cervical, 50–69 for breast, and 50 and above for colorectal. ^¢^CHS 2016 age groups were 40–69 for cervical screening questions. ^╤^The difference in proportion between knowledge of the cancer screening test and ever screened with the test.

Supplementary Table 4 Cancer screening knowledge and participation rates, stratified by family history.

Supplementary Figure 1 Age distribution in the survey sample in comparison to Singapore’s population. **Based on Singapore SingStat 2016 Population Data ages 40 and above.

Supplementary Figure 2 Gender distribution in the survey sample in comparison to Singapore’s population. **Based on Singapore SingStat 2016 Population Data ages 40 and above.

Supplementary Figure 3 Ethnic distribution in the survey sample in comparison to Singapore’s population. **Based on Singapore SingStat 2016 Population Data ages 40 and above.

## Abbreviations

aPR, adjusted prevalence ratio; CI, confidence interval; HPB, Health Promotion Board; FOBT, faecal occult blood test; NUS, National University of Singapore; SSHSPH, Saw Swee Hock School of Public Health.
