# Supplementary material for: Cancer Screening Knowledge and Behavior in a Multi-Ethnic Asian Population: The Singapore Community Health Study
Source: Front Oncol. 2021 Aug 12;11:684917. doi: 10.3389/fonc.2021.684917 (PMC8406849; doi:10.3389/fonc.2021.684917)
Supplement: Supplementary Table 1 — Adjusted prevalence ratio (aPR) estimates for characteristics associated with those tested without knowledge of Pap smear. Multivariate modified Poisson regression model analyses were adjusted for age, ethnicity, education, monthly household income, housing type, and living arrangement. *Based on recommended screening guideline for cervical cancers as defined by MOH guidelines: Pap smear for sexually active females aged 25 to 69 years at least once every 3 years. [file Table_1.docx]

**Supplemental Table 1. Adjusted prevalence ratio (aPR) estimates for characteristics associated with those tested without knowledge of Pap smear**

|  | **Those who do not know what a Pap smear is but did the test*** | |
| --- | --- | --- |
|  | n = 318 (44.7%) | |
| Characteristic | aPR (95% CI) | p-value |
| Age(years) |  | |
| 40-49 | Ref | |
| 50-59 | 0.91(0.73-1.15) | 0.45 |
| 60-69 | 0.95(0.74-1.21) | 0.67 |
| Ethnicity |  | |
| Chinese | Ref | |
| Malay | 0.45(0.27-0.75) | 0.002 |
| Indian | 0.36(0.16-0.82) | 0.015 |
| Others | 0.48(0.14-1.57) | 0.22 |
| Education |  | |
| Primary and below | Ref | |
| Lower secondary | 1.28(1.04-1.58) | 0.018 |
| Secondary | 0.98(0.72-1.34) | 0.92 |
| Junior College | 1.10(0.68-1.77) | 0.70 |
| Polytechnic/Arts Institution | 1.31(0.89-1.94) | 0.17 |
| University & above | 0.71(0.35-1.45) | 0.35 |
| Monthly household income ($S) |  | |
| < $2,000 | Ref | |
| $2,000-$3,999 | 1.32(1.07-1.63) | 0.010 |
| $4,000-$5,999 | 1.41(1.09-1.83) | 0.009 |
| $6,000-$9,999 | 0.95(0.61-1.49) | 0.84 |
| ≥$10,000 | 1.34(0.65-2.77) | 0.43 |
| Housing type |  | |
| ≤2-room public flat | Ref | |
| 3-room public flat | 1.13(0.73-1.73) | 0.59 |
| ≥4-room public flat/private | 1.42(0.94-2.16) | 0.098 |
| Living arrangement |  | |
| Alone | Ref | |
| With others | 1.15(0.71-1.85) | 0.58 |

Multivariate modified Poisson regression model analyses were adjusted for age, ethnicity, education, monthly household income, housing type, and living arrangement.

*Based on recommended screening guideline for cervical cancers as defined by MOH guidelines: Pap smear for sexually active females aged 25 to 69 years at least once every 3 years.
