# Supplementary material for: Cancer Screening Knowledge and Behavior in a Multi-Ethnic Asian Population: The Singapore Community Health Study
Source: Front Oncol. 2021 Aug 12;11:684917. doi: 10.3389/fonc.2021.684917 (PMC8406849; doi:10.3389/fonc.2021.684917)
Supplement: Supplementary Table 2 — Adjusted prevalence ratio (aPR) estimates for characteristics associated with screening as recommended for all three cancers. Multivariate modified Poisson regression model analyses were adjusted for age, ethnicity, education, monthly household income, housing type, living arrangement, past history of any cancer, family history of any cancer, and frequent smoking. *Based on recommended screening guidelines for selected cancers as defined by MOH guidelines: cervical cancer—Pap smear for sexually active females aged 25 to 69 years at least once every 3 years; breast cancer—mammography for females aged 50 to 69 years every 2 years; colorectal cancer–faecal occult blood test (FOBT) done annually or sigmoidoscopy/colonoscopy once every 10 years for individuals aged ≥50 years. aFrequent smoking is defined as smoking cigarettes daily. [file Table_2.docx]

**Supplemental Table 2. Adjusted prevalence ratio (aPR) estimates for characteristics associated with screening as recommended for all three cancers**

|  | Women aged 50-69 who had screened as recommended* for all three cancers | |
| --- | --- | --- |
|  | n = 272 (10.7%) | |
| Characteristic | aPR (95% CI) | p-value |
| Age(years) |  | |
| 50-59 | Ref | |
| 60-69 | 1.02 (0.78-1.34) | 0.89 |
| Ethnicity |  | |
| Chinese | Ref | |
| Malay | 0.53 (0.33-0.84) | 0.008 |
| Indian | 0.97 (0.64-1.49) | 0.90 |
| Others | 1.19 (0.50-2.84) | 0.69 |
| Education |  | |
| Primary and below | Ref | |
| Lower secondary | 1.42 (0.96-2.11) | 0.079 |
| Secondary | 1.73 (1.21-2.47) | 0.002 |
| Junior College | 1.33 (0.73-2.42) | 0.35 |
| Polytechnic/Arts Institution | 1.61 (0.99-2.61) | 0.055 |
| University & above | 1.36 (0.78-2.38) | 0.28 |
| Monthly household income ($S) |  | |
| < $2,000 | Ref | |
| $2,000-$3,999 | 1.11 (0.76-1.62) | 0.57 |
| $4,000-$5,999 | 1.41 (0.94-2.09) | 0.093 |
| $6,000-$9,999 | 2.06 (1.38-3.07) | <0.001 |
| ≥$10,000 | 2.45 (1.53-3.92) | <0.001 |
| Housing type |  | |
| ≤2-room public flat | Ref | |
| 3-room public flat | 3.03 (0.75-12.27) | 0.12 |
| ≥4-room public flat/private | 3.58 (0.92-14.04) | 0.067 |
| Living arrangement |  | |
| Alone | Ref | |
| With others | 1.54 (0.74-3.17) | 0.25 |
| Past history of any cancer |  | |
| No | Ref | |
| Yes | 1.63 (1.05-2.52) | 0.028 |
| Family history of any cancer |  | |
| No | Ref | |
| Yes | 1.12 (0.88-1.43) | 0.37 |
| Frequent smoking^a^ |  | |
| No | Ref | |
| Yes | 1.39 (0.59-3.26) | 0.45 |

Multivariate modified Poisson regression model analyses were adjusted for age, ethnicity, education, monthly household income, housing type, living arrangement, past history of any cancer, family history of any cancer, and frequent smoking.

*Based on recommended screening guidelines for selected cancers as defined by MOH guidelines: cervical cancer - Pap smear for sexually active females aged 25 to 69 years at least once every 3 years; breast cancer - mammography for females aged 50 to 69 years every 2 years; colorectal cancer - faecal occult blood test (FOBT) done annually or sigmoidoscopy/colonoscopy once every 10 years for individuals aged ≥50 years.

^a^Frequent smoking is defined as smoking cigarettes daily.
