# Supplementary material for: Cancer Screening Knowledge and Behavior in a Multi-Ethnic Asian Population: The Singapore Community Health Study
Source: Front Oncol. 2021 Aug 12;11:684917. doi: 10.3389/fonc.2021.684917 (PMC8406849; doi:10.3389/fonc.2021.684917)
Supplement: Supplementary Table 3 — Cancer screening participation rates in Singapore. NHS National Health Survey; CHS, Community Health Survey. Unless otherwise stated, the screening questions involved age groups 25–69 for cervical, 50–69 for breast, and 50 and above for colorectal. ¢CHS 2016 age groups were 40–69 for cervical screening questions. ╤The difference in proportion between knowledge of the cancer screening test and ever screened with the test. [file Table_3.docx]

**Supplemental Table 3. Cancer screening participation rates in Singapore**

|  | **NHS**  **2004** | **NHS**  **2010** | **CHS**  **2016** |  |
| --- | --- | --- | --- | --- |
| Cervical | 80.8% | 87.1% | 80.2%^₵^ | Knowledge of Pap Smear |
|  | 70.1% | 71.3% | 77.1%^₵^ | Ever had Pap Smear |
|  | 10.7% | 15.8% | 3.1%^₵^ | Knowledge-behaviour gap^₸^ |
|  | 52.0% | 47.9% | 43.0%^₵^ | Screened as recommended |
| Breast | 79.9% | 90.9% | 93.6% | Knowledge of Mammography |
|  | 54.2% | 66.3% | 75.2% | Ever had Mammogram |
|  | 25.7% | 24.6% | 18.4% | Knowledge-behaviour gap^₸^ |
|  | 39.2% | 39.6% | 35.0% | Screened as recommended |
| Colorectal | 17.3% | 27.8% | 42.9% | Ever had FOBT |
|  | 11.2% | 14.2% | 22.1% | Ever had sigmoidoscopy/colonoscopy |
|  | - | 36.3% | 49.0% | Ever had FOBT/sigmoidoscopy/colonoscopy |
|  | - | 20.2% | 27.3% | Screened as recommended |

NHS = National Health Survey, CHS = Community Health Survey

Unless otherwise stated, the screening questions involved age groups 25-69 for cervical, 50-69 for breast, and 50 and above for colorectal.

^₵^CHS 2016 age groups were 40-69 for cervical screening questions.

^₸^The difference in proportion between knowledge of the cancer screening test and ever screened with the test.
