# Supplementary material for: Cancer Screening Knowledge and Behavior in a Multi-Ethnic Asian Population: The Singapore Community Health Study
Source: Front Oncol. 2021 Aug 12;11:684917. doi: 10.3389/fonc.2021.684917 (PMC8406849; doi:10.3389/fonc.2021.684917)
Supplement: Supplementary Table 4 — Cancer screening knowledge and participation rates, stratified by family history. [file Table_4.docx]

**Supplemental Table 4. Cancer screening knowledge and participation rates, stratified by family history**

|  | Number of respondents eligible for screening as recommended | Reported having knowledge of screening test^₸^ | Those who had ever been screened | Those who had screened as recommended* |
| --- | --- | --- | --- | --- |
|  | Total (N) | n(%) | n(%) | n(%) |
| **Pap Smear** | 3584 | 2872 (80.0) | 2763 (77.2) | 1539 (43.0) |
| No FHx of any cancer | 2344 | 1854 (79.1) | 1780 (75.9) | 990 (42.2) |
| Yes FHx of any cancer | 1240 | 1019 (82.2) | 984 (79.4) | 550 (44.4) |
| **Mammography** | 2532 | 2370 (93.6) | 1903 (75.2) | 889 (35.1) |
| No FHx of any cancer | 1602 | 1481 (92.5) | 1171 (73.1) | 545 (34.0) |
| Yes FHx of any cancer | 930 | 889 (95.6) | 732 (78.7) | 342 (36.8) |
| **FOBT only** | 5281 | - | 2267 (42.9) | - |
| No FHx of any cancer | 3551 | - | 1437 (40.5) | - |
| Yes FHx of any cancer | 1730 | - | 830 (48.0) | - |
| **Colonoscopy/ Sigmoidoscopy only** | 5281 | - | 1167 (22.1) | - |
| No FHx of any cancer | 3551 | - | 700 (19.7) | - |
| Yes FHx of any cancer | 1730 | - | 467 (27.0) | - |
| **FOBT/Colonoscopy/ Sigmoidoscopy** | 5281 | - | 2589 (49.0) | 1440 (27.3) |
| No FHx of any cancer | 3551 | - | 1632 (46.0) | 874 (24.6) |
| Yes FHx of any cancer | 1730 | - | 957 (55.3) | 566 (32.7) |
| **All of the above°** | 2536 | - | - | 272 (10.7) |
| No FHx of any cancer | 1604 | - | - | 152 (9.5) |
| Yes FHx of any cancer | 932 | - | - | 120 (12.9) |

*Based on recommended screening guidelines for selected cancers as defined by MOH guidelines:

cervical cancer - Pap smear for sexually active females aged 25 to 69 years at least once every 3 years; breast cancer - mammography for females aged 50 to 69 years every 2 years; colorectal cancer - faecal occult blood test (FOBT) done annually or sigmoidoscopy/colonoscopy once every 10 years for individuals aged ≥50 years

^₸^Due to limitations of the collected data, knowledge for colorectal cancer screening was not reported.

^°^Pap Smear, Mammography, and either FOBT or Colonoscopy/Sigmoidoscopy
