# Supplementary figures and images for: Cancer Screening Knowledge and Behavior in a Multi-Ethnic Asian Population: The Singapore Community Health Study
Source: Front Oncol. 2021 Aug 12;11:684917. doi: 10.3389/fonc.2021.684917 (PMC8406849; doi:10.3389/fonc.2021.684917)

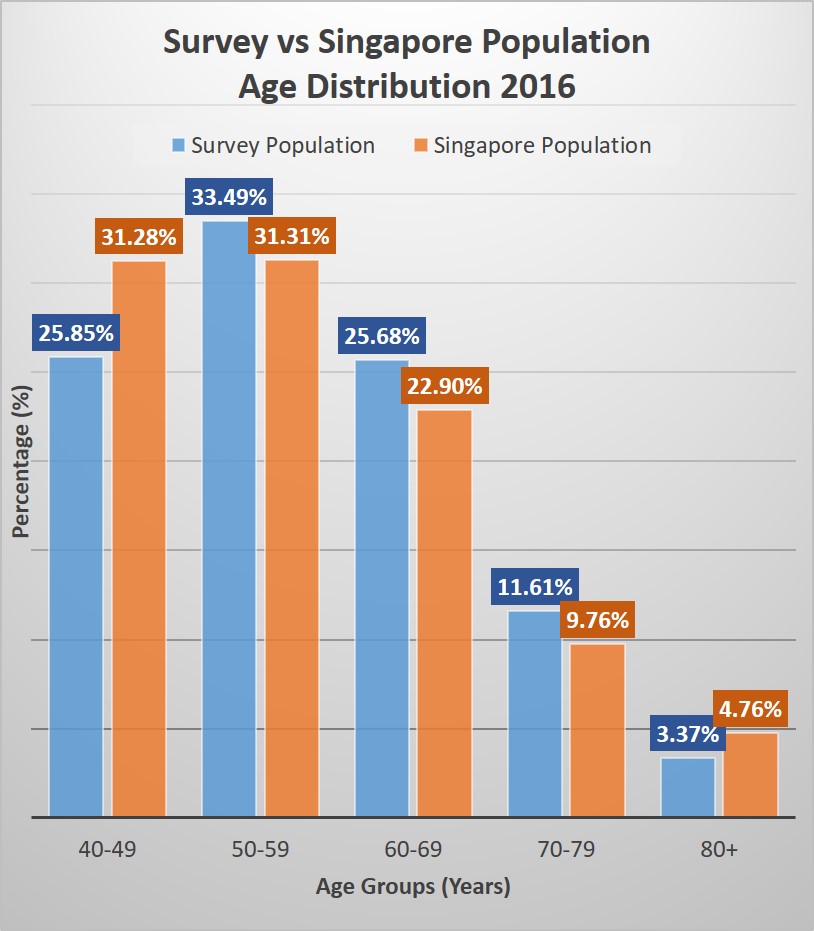

Supplement: Supplementary Figure 1 — Age distribution in the survey sample in comparison to Singapore’s population. **Based on Singapore SingStat 2016 Population Data ages 40 and above. [file Image_1.jpg]

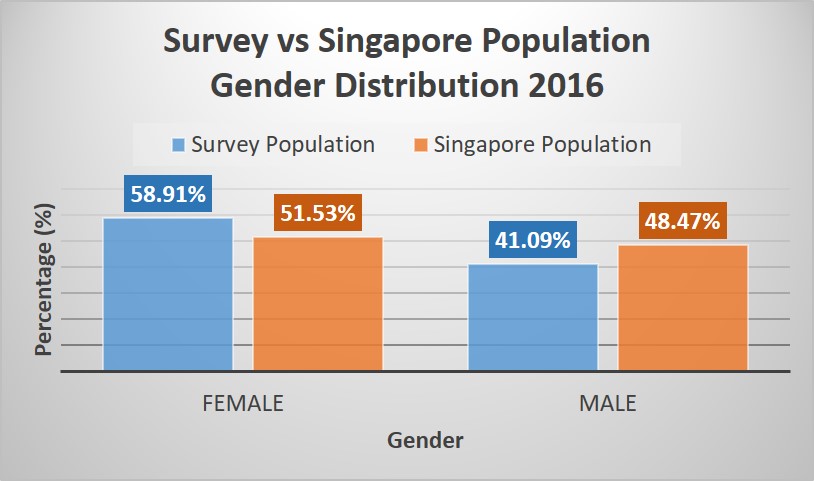

Supplement: Supplementary Figure 2 — Gender distribution in the survey sample in comparison to Singapore’s population. **Based on Singapore SingStat 2016 Population Data ages 40 and above. [file Image_2.jpg]

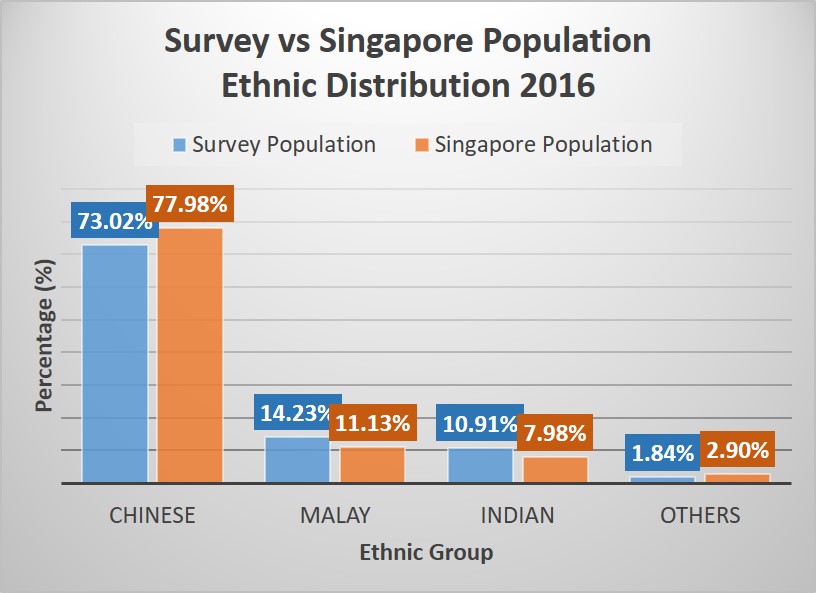

Supplement: Supplementary Figure 3 — Ethnic distribution in the survey sample in comparison to Singapore’s population. **Based on Singapore SingStat 2016 Population Data ages 40 and above. [file Image_3.jpg]
